# Mixed Pancreatobiliary Ductal Adenocarcinoma and Squamous Cell Carcinoma Arising from an Ectopic Pancreas in a Gastric Duplication Cyst—A Rare Double Diagnosis

**DOI:** 10.3390/diagnostics14232727

**Published:** 2024-12-04

**Authors:** Minhye Kim, Jungwook Yang, Daehyun Song, Hyojung An, Dongchul Kim

**Affiliations:** 1 Department of Pathology, Gyeongsang National University Hospital, Jinju 52727, Republic of Korea; joymine86@naver.com (M.K.); woogi1982@gnu.ac.kr (J.Y.); 2 Department of Pathology, Gyeongsang National University School of Medicine, Jinju 52727, Republic of Korea; daehyun@gnu.ac.kr (D.S.); ariel2020@naver.com (H.A.); 3 Institute of Medical Science, Gyeongsang National University, Jinju 52727, Republic of Korea; 4 Department of Pathology, Gyeongsang National University Changwon Hospital, Changwon 51472, Republic of Korea

**Keywords:** adenocarcinoma, gastric duplication cysts, ectopic pancreas

## Abstract

Gastric duplication cysts (GDCs) are rare congenital anomalies, often identified during infancy or childhood. Although typically benign, there have been sporadic reports of malignant transformations, including adenocarcinoma and rare mixed tumors. Herein, we describe a rare case of mixed pancreatobiliary ductal adenocarcinoma and squamous cell carcinoma occurring within a GDC in a 54-year-old Korean woman with a history of melena and hematemesis. Initial gastroscopy and positron emission tomography–computed tomography (PET-CT) revealed a protruding stomach mass. A laparoscopic total gastrectomy was performed, and histological examination confirmed a mixed carcinoma originating from an ectopic pancreas within the duplication cyst. This case is unique as it is the first reported instance in the world of mixed pancreatobiliary ductal adenocarcinoma and squamous cell carcinoma arising from an ectopic pancreas within a GDC. This highlights the importance of considering pancreatobiliary-type adenocarcinoma in the differential diagnosis of malignancies originating from GDCs, which has implications for treatment strategies.

**Figure 1:**
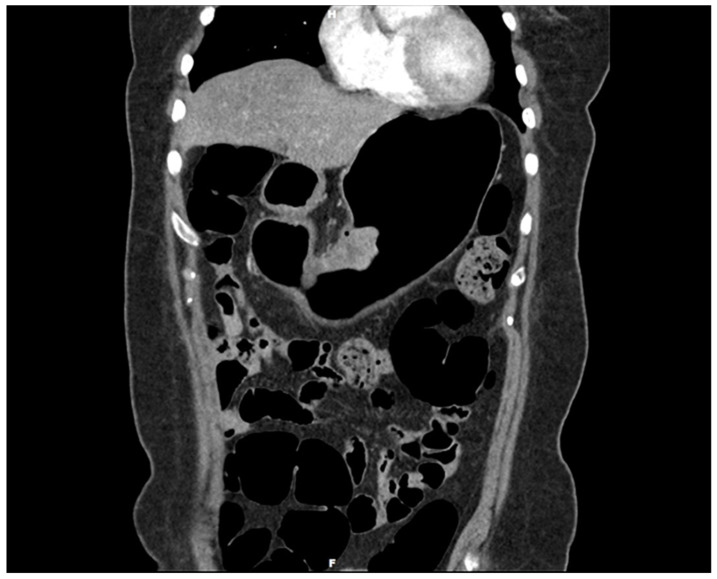
Gastroscopy with three-dimensionally enhanced computed tomography (3D ECT) showing a protruding tumor in the mid-body on the lesser curvature. Gastric duplication cysts (GDCs) are rare congenital anomalies first described in 1911 by Wendel and typically identified in infancy or childhood. Although the exact pathogenesis remains elusive, these cysts are believed to result from aberrations during embryonic development of the foregut [1,2,3]. Characteristically, they share a common wall with the stomach and possess an epithelial lining similar to the gastrointestinal tract [4]. The clinical presentation of GDCs can vary significantly, ranging from asymptomatic to severe abdominal symptoms, depending on their size, location, and the presence of associated complications. Although most GDCs are benign, there have been sporadic reports of malignant transformations within these cysts. Adenocarcinoma is the most commonly reported malignancy arising from GDCs, as evidenced by case reports and small case series [5,6,7,8,9]. Moreover, mixed tumors arising from GDCs are rare. To date, only two cases of mixed adenocarcinoma and squamous cell carcinoma arising from GDCs have been reported [10,11]. Herein, we describe a rare case of mixed adenocarcinoma and squamous cell carcinoma that developed within a GDC. However, it differs from the previous report in that the adenocarcinoma component was pancreatobiliary-type ductal adenocarcinoma. Moreover, this case report is the first to report a mixed pancreatobiliary ductal adenocarcinoma and squamous cell carcinoma in a GDC. It contributes a novel aspect to the scientific understanding of the malignant potential of GDCs and underscores the necessity for vigilance in managing these rare entities. By examining this case, we aim to enrich the discourse on GDCs, particularly their neoplastic capabilities, and provide insights into more effective diagnostic and therapeutic strategies. A 54-year-old Korean woman presented with melena that began a month ago and vomiting with hematemesis over the last 3 days. The patient had no other medical, surgical, or significant personal or family histories. She visited a local hospital for assessment and underwent gastroscopy. Gastroscopy revealed a protruding mass with bleeding in the stomach, which was suggestive of malignancy. The local hospital could not perform a biopsy due to the bleeding, and the patient visited our hospital for further evaluation. A 3D ECT scan revealed a protruding mass in the lower gastric body and enlarged perigastric lymph nodes (this figure). An esophagogastroduodenoscopy revealed an approximately 5 cm-sized protruding mass in the mid-body of the lesser curvature. Endoscopic biopsy revealed a malignancy suggestive of mixed adenocarcinoma and squamous cell carcinoma. PET-CT detected a 5 cm-sized focal hypermetabolic activity in the body of the stomach and focal increased fluorodeoxyglucose uptake in the perigastric lymph nodes; otherwise, the findings were unremarkable. Upon laboratory examination, the results of the complete blood count and routine biochemical investigations were unremarkable. Blood tumor marker tests showed elevated levels of carcinoembryonic antigen (CEA) (52.2 ng/mL; normal range: 0.0–3.4 ng/mL) and carbohydrate antigen 19-9 (CA19-9) (317.0 U/mL; normal range: 0–34 U/mL).

**Figure 2:**
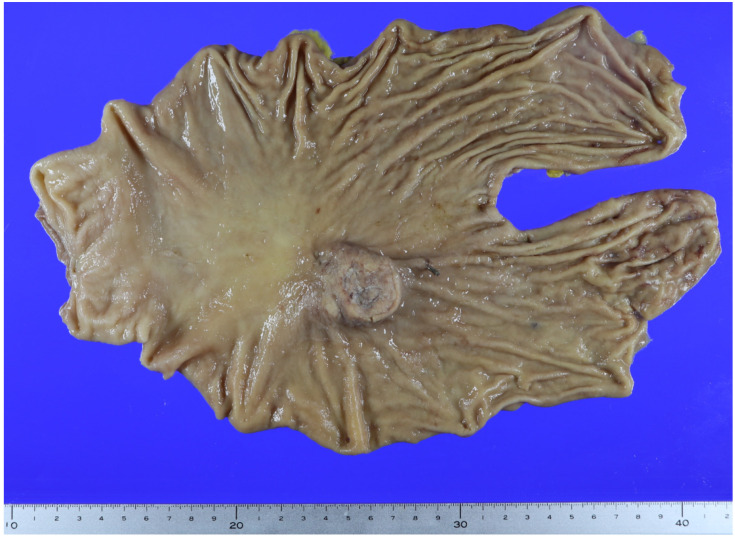
A gross image of the gastrectomy specimen. The patient underwent a total laparoscopic gastrectomy. This specimen shows a protruding tumor in the mid-body on the lesser curvature, measuring 50 × 45 mm. Sectioning of the mass revealed cystic changes.

**Figure 3:**
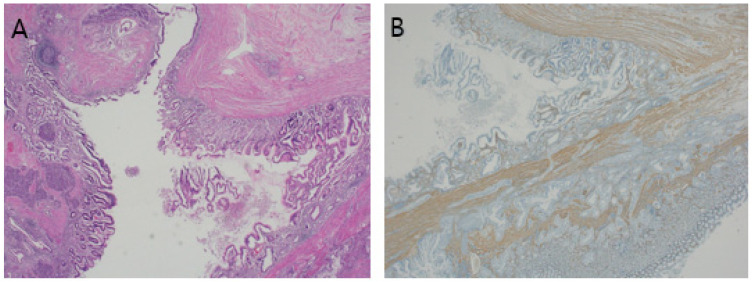
Gastric duplication cyst. (**A**) Within the cystic mass, inner gastric-type mucosa and muscular layer are observed. An ectopic pancreas is present. (**B**) Staining for smooth muscle actin shows the sharing of the proper muscle between the duplication cyst and stomach (×40, original magnification). Gastric duplication cysts possess a well-developed layer of smooth muscle and an epithelial lining that resembles part of the gastrointestinal tract. These cysts are attached to the stomach and share a common muscle wall [6]. Microscopically, the stomach epithelium did not show any malignant changes. A cystic mass was observed in the proper muscle; within the cystic mass, the inner layer was gastric-type mucosa. The muscular layer and an ectopic pancreas were observed. Staining for smooth muscle actin protein revealed that the proper muscle of the cyst was shared with that of the stomach. Therefore, we diagnosed the patient with a GDC.

**Figure 4:**
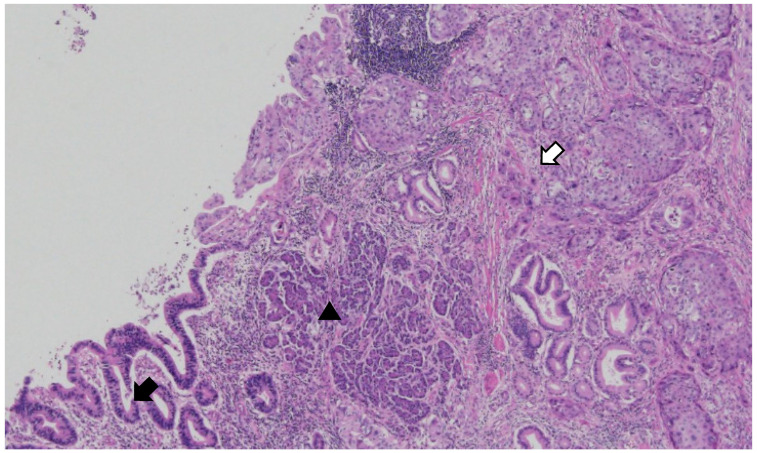
Mixed adenocarcinoma and squamous cell carcinoma within the duplication cyst. The tumor originates from the ectopic pancreas in the duplication cyst. (Black arrow: adenocarcinoma; white arrow: squamous carcinoma; arrow head: ectopic pancreas) (×40, original magnification). The carcinoma was present in the cystic portion of the proper stomach muscle and had invaded the mucosal layer. Pancreatic tissue was present in the proper muscle layer of the cystic lesion, and the carcinoma originated from the ductal epithelium of the ectopic pancreas. Of the two types of malignant tumors, adenocarcinoma and squamous cell carcinoma, the adenocarcinoma component accounted for approximately 55%, and the squamous cell carcinoma component accounted for approximately 45%. Surgical resection margins tested negative for tumor cells. Lymphovascular and neural invasions were observed. Lymph node (LN) metastasis was observed in 6 of 25 perigastric LNs. In metastatic LNs, the squamous component was predominant.

**Figure 5:**
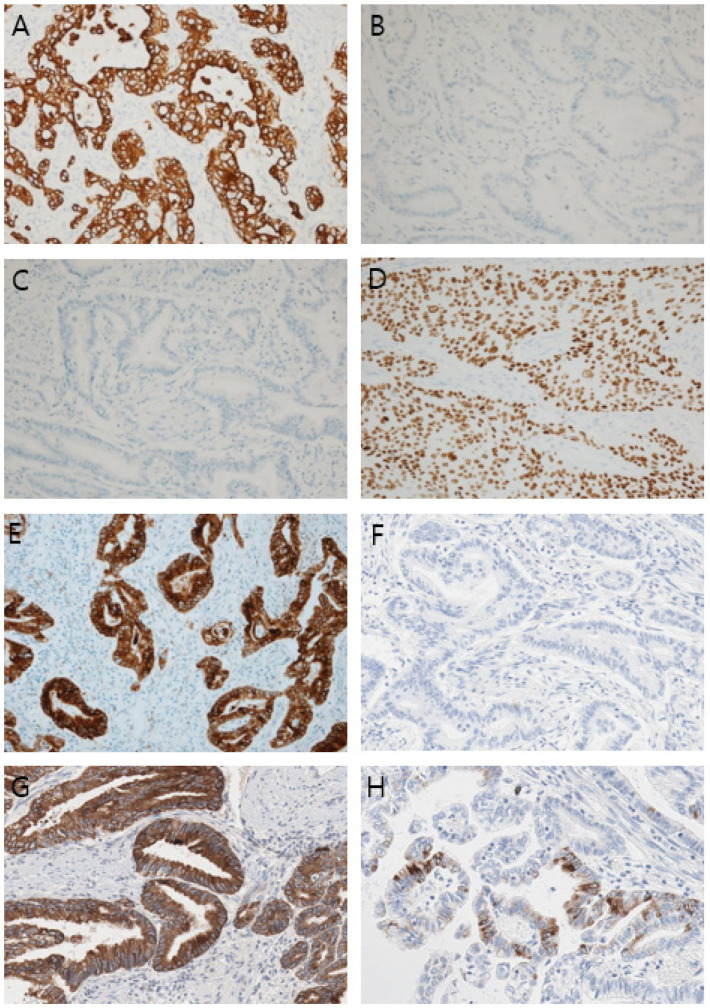
Results of an immunohistochemical study (×100, original magnification). (**A**) Cytokeratin 7, (**B**) Cytokeratin 20, (**C**) CDX2, (**D**) p40, (**E**) Mucin (MUC)1, (**F**) MUC2, (**G**) MUC5AC, (**H**) MUC6. The mucin expression profile was positive for MUC1 and MUC5AC, patchy for MUC6, and negative for MUC2. The results indicate that the adenocarcinoma portion is pancreatobiliary-type. Immunohistochemical analysis of the cancer cells showed diffuse strong positivity for cytokeratin 7 in the adenocarcinoma and squamous cell carcinoma components and negative results for cytokeratin 20 and CDX2. p40 showed diffuse positivity in the squamous cell carcinoma component. The mucin expression profile was positive for mucin (MUC)1 and MUC5AC, patchy for MUC6, and negative for MUC2 (this figure). Herein, the final diagnosis was mixed pancreatobiliary-type ductal adenocarcinoma and squamous cell carcinoma arising from an ectopic pancreas in a duplication cyst in the stomach [12]. The patient received chemotherapy with a fixed eight-cycle oxaliplatin and capecitabine (XELOX) regimen after surgery. Two years after chemotherapy, CEA (3.77 ng/mL) and CA19-9 (16.9 U/mL) levels returned to almost normal, and there has been no relapse after 28 months. This case demonstrates a particularly rare occurrence arising from a gastrointestinal duplication cyst (GDC), a type of congenital malformation where a portion of the gastrointestinal tract duplicates and attaches to another part of the tract. Although the pathogenesis of GDCs is not fully understood, the most widely accepted theory attributes their formation to improper or incomplete recanalization during fetal development [13]. The cyst is usually asymptomatic but can cause symptoms such as upper abdominal obstruction, abdominal pain, and a palpable mass, particularly in infancy. Symptoms vary according to the cyst size and the location [14]. The potential complications include gastrointestinal bleeding, gastric perforation, and torsion. In rare cases, it can progress to malignancy [13,15]. Adenocarcinoma is the most common malignancy of GDC [2,5,6,7,8,9]. Other types have been reported in the literature, including mixed adenocarcinoma and squamous cell carcinoma, sarcomatoid carcinoma, neuroendocrine carcinoma, and gastrointestinal stromal tumor [10,11,16,17,18]. Most adenocarcinomas diagnosed are of the gastric type, and only one case has been reported of the pancreatobiliary type [19]. Two cases of mixed adenocarcinoma and squamous cell carcinoma have been reported from a GDC [10,11]. Our patient also had mixed adenocarcinoma and squamous cell carcinoma, as reported above. However, while the adenocarcinoma in the previous two cases was a general gastric type, which, in our patient, was confirmed to be of the pancreatobiliary type through an immunohistochemical study [12]. Moreover, the tumor in our patient appeared to have originated from a carcinoma in the ectopic pancreas in a duplication cyst. As a result, our patient had a mixed pancreatobiliary-type adenocarcinoma and squamous cell carcinoma arising from the ectopic pancreas within a GDC. In duplication cysts, ectopic pancreas is present in up to 10% of cases, and complications such as pancreatitis or pancreatic pseudocysts [20] have been reported. To our knowledge, malignancy, which originates from the ectopic pancreas in a gastric duplication cyst, has not been reported. Malignant transformation in the ectopic pancreas alone is very rare, with an incidence of 0.7–1.8% [21,22]. Therefore, our case report concerns an extremely rare case and the first report of a double pancreatobiliary-type adenocarcinoma among the complex tumors of a GDC and the first malignant tumor arising from an ectopic pancreas in a GDC. A limitation of this study was the inability to further investigate genetic mutations in the samples. While diagnosing a condition based on microscopic findings and immunohistochemical study results poses no significant difficulty, encountering a rare case of this nature in the future warrants the validation of genetic information through tests such as next-generation sequencing. Treatment approaches for adenocarcinoma, mixed adenocarcinoma, and squamous cell carcinoma differ because of their different pathological characteristics. Moreover, even for the same adenocarcinoma, the drugs used vary depending on the type [23,24,25,26]. After surgery, our patient underwent chemotherapy based on the treatment regimen for mixed adenocarcinoma and squamous carcinoma of the stomach. As of 28 months post-treatment, no recurrence has been observed. Given the rarity of this condition, there are no established treatment guidelines. We also suggest that treatment regimens typically used for pancreatobiliary adenocarcinoma should be considered for similar cases. In conclusion, we report the first case of mixed pancreatobiliary adenocarcinoma and squamous cell carcinoma of an ectopic pancreas in a duplication stomach cyst.

## Data Availability

The datasets used and/or analyzed in the current study are available from the corresponding author upon reasonable request.

## References

[1] García Nebreda M., Paseiro Crespo G., Álvaro Cifuentes E., Marqués Medina E., Burdaspal Moratilla A. (2019). Gastric duplication cyst with respiratory epithelium: An uncommon injury that has a difficult differential diagnosis. Cir. Esp. (Engl. Ed.).

[2] Kinugasa S., Monma H., Sakamoto Y., Watanabe T., Fujimoto M. (2020). Adenocarcinoma arising from a gastric duplication cyst with lymph node metastasis. Cureus.

[3] Kim D.H., Kim J.S., Nam E.S., Shin H.S. (2000). Foregut duplication cyst of the stomach. Pathol. Int..

[4] Singh J.P., Rajdeo H., Bhuta K., Savino J.A. (2013). Gastric duplication cyst: Two case reports and review of the literature. Case Rep. Surg..

[5] Abdulla M.A.M., Al Saeed M., Ameer Alshaikh S., Nabar U.J. (2017). Adenocarcinoma arising from a gastric duplication cyst: A case report and literature review. Int. Med. Case Rep. J..

[6] Chan B.P.H., Hyrcza M., Ramsay J., Tse F. (2018). Adenocarcinoma Arising from a Gastric Duplication Cyst. ACG Case Rep. J..

[7] Kaneko T., Ohara M., Okamura K., Fujiwara-Kuroda A., Miyasaka D., Yamabuki T., Takahashi R., Komuro K., Suzuoki M., Iwashiro N. (2019). Adenocarcinoma arising from an ectopic pancreas in the duodenum: A case report. Surg. Case Rep..

[8] Kang H.J., Jang S.J., Park Y.S. (2014). Adenocarcinoma arising in gastric duplication cyst. Korean J. Pathol..

[9] Losefsky Q.P., Cho E., Jeyarajah D.R. (2022). Adenocarcinoma arising in a gastric duplication cyst. J. Gastrointest. Surg..

[10] Barussaud M.L., Meurette G., Cassagnau E., Dupas B., Le Borgne J. (2008). Mixed adenocarcinoma and squamous cell carcinoma arising in a gastric duplication cyst. Gastroenterol. Clin. Biol..

[11] Han S.Y., Kim G.H. (2017). Collision tumor arising from a gastric duplication cyst. Gastrointest. Endosc..

[12] Wang S., You L., Dai M., Zhao Y. (2020). Mucins in pancreatic cancer: A well-established but promising family for diagnosis, prognosis and therapy. J. Cell. Mol. Med..

[13] Puligandla P.S., Nguyen L.T., St-Vil D., Flageole H., Bensoussan A.L., Nguyen V.H., Laberge J.M. (2003). Gastrointestinal duplications. J. Pediatr. Surg..

[14] Kuraoka K., Nakayama H., Kagawa T., Ichikawa T., Yasui W. (2004). Adenocarcinoma arising from a gastric duplication cyst with invasion to the stomach: A case report with literature review. J. Clin. Pathol..

[15] Van Pham N., Van Mai D., Duong P.D.T., Lam H.H., Ly H.H.V., Van Nguyen L. (2024). Duplication cyst in adult cases: A journey from diagnosis to treatment. J. Surg. Case Rep..

[16] Ahmed M.A.H., Liyanaarachchi K.S., Preston S.R., Hewish M., Bagwan I.N. (2021). Sarcomatoid carcinoma arising in a gastric duplication cyst. ACG Case Rep. J..

[17] Fernandez D.C., Machicado J., Davogustto G. (2016). Gastrointestinal stromal tumor arising from a gastric duplication cyst. ACG Case Rep. J..

[18] Horne G., Ming-Lum C., Kirkpatrick A.W., Parker R.L. (2007). High-grade neuroendocrine carcinoma arising in a gastric duplication cyst: A case report with literature review. Int. J. Surg. Pathol..

[19] Rolo A., Oliveira R.C., Lima B., Barbosa A., Faustino I. (2021). Pancreatobiliary adenocarcinoma in a gastric duplication cyst: A doubly rare diagnosis. Cureus.

[20] Passos I.D., Chatzoulis G., Milias K., Tzoi E., Christoforakis C., Spyridopoulos P. (2017). Gastric duplication cyst (gdc) associated with ectopic pancreas: Case report and review of the literature. Int. J. Surg. Case Rep..

[21] Cazacu I.M., Luzuriaga Chavez A.A., Nogueras Gonzalez G.M., Saftoiu A., Bhutani M.S. (2019). Malignant transformation of ectopic pancreas. Dig. Dis. Sci..

[22] Nagtegaal I.D., Odze R.D., Klimstra D., Paradis V., Rugge M., Schirmacher P., Washington K.M., Carneiro F., Cree I.A., WHO Classification of Tumours Editorial Board (2020). The 2019 WHO classification of tumours of the digestive system. Histopathology.

[23] Springfeld C., Jäger D., Büchler M.W., Strobel O., Hackert T., Palmer D.H., Neoptolemos J.P. (2019). Chemotherapy for pancreatic cancer. Presse Med..

[24] Wagner A.D., Syn N.L., Moehler M., Grothe W., Yong W.P., Tai B., Ho J., Unverzagt S. (2017). Chemotherapy for advanced gastric cancer. Cochrane Database Syst. Rev..

[25] Wld A.T., Dholakia A.S., Fan K.Y., Kumar R., Moningi S., Rosati L.M., Laheru D.A., Zheng L., De Jesus-Acosta A., Ellsworth S.G. (2015). Efficacy of platinum chemotherapy agents in the adjuvant setting for adenosquamous carcinoma of the pancreas. J. Gastrointest. Oncol..

[26] Li H.S., Liu X., Zhang M.Y., Cheng K., Chen Y., Zhou Y.W., Liu J.Y. (2020). Clinicopathologic characteristics, survival, and treatments for gastric adenosquamous carcinoma: A population-based study. Curr. Oncol..

